# IL-33/IL1RL1 (ST2) signalling drives immune suppression in squamous intraepithelial hyperplasia

**DOI:** 10.1016/j.tvr.2026.200350

**Published:** 2026-09-01

**Authors:** Chenhao Zhou, Jazmina Gonzalez Cruz, Siok Min Teoh, Minh Tran, Denise Goh, Divyaa Narayanan, Xiao Tan, Thi Viet Trinh Dang, Zewen Kelvin Tuong, Kevin Gillinder, Abate Bashaw, James W. Wells, Ian H. Frazer, Quan Nguyen, Yu Chen, Meihua Yu, Janin Chandra

**Affiliations:** a Frazer Institute, Faculty of Health, Medicine, and Behavioural Sciences, The University of Queensland, Brisbane, Queensland, 4102, Australia; b Dermatology Research Centre, The University of Queensland, Brisbane, Queensland, 4102, Australia; c Institute for Molecular Bioscience, The University of Queensland, St Lucia, Queensland, 4067, Australia; d QIMR Berghofer Medical Research Institute, Brisbane, Australia; e Materdicine Lab, School of Life Sciences, Shanghai University, Shanghai, 200444, PR China

## Abstract

IL-33, an alarmin cytokine binding to IL1RL1 (ST2) receptor, plays a multifaceted role in cancer. Using an HPV16-E7 oncoprotein-mediated murine model of squamous intraepithelial hyperplasia, a pre-stage of squamous cell carcinoma (SCC), we characterised the interactions of hyperproliferative epithelial cells and immune cells regulated by IL-33 and IL1RL1. We show that basal epithelial cells in epithelial hyperplasia co-express IL-33 and genes involved in MHC class II antigen presentation and in epithelial stress response. Using spatial transcriptomics, we demonstrate that IL1RL1^+^Foxp3^+^Tregs are in close proximity to IL-33-expressing cells, in both the murine model and in human cervical intraepithelial neoplastic tissues. Cytoplasmic location of IL-33 is associated with its bioactivity, and we show that a subset of epithelial cells in epithelial hyperplasia contains high levels of cytoplasmic IL-33. Genetic deletion of the IL1RL1 gene led to partial rejection of HPV16-E7-expressing skin grafts, demonstrating that IL1RL1 plays a functional role in mediating immune suppression in epithelial hyperplasia. IL1RL1-deletion further led to a significant reduction in Tregs in epithelial hyperplasia. The IL1RL1 signalling pathway, therefore, represents a potential therapeutic target for intraepithelial hyperplasia and SCC.

## 1 Introduction

Epithelial cancers account for approximately 90% of human malignancies, of which squamous cell carcinomas (SCCs) represent an archetype that displays cardinal features of the immune microenvironment. SCCs include malignancies of the skin, the oral cavity and throat, the oesophagus and those of anogenital tissues, including the cervix, vagina, vulva, penis and anus. Certain SCCs are associated with persistent infection with oncogenic human papillomavirus (HPV) genotypes, such as 99.7% of cervical SCCs [1] and 30-70% of head and neck SCCs [2]. Immune checkpoint inhibition therapy has had limited success for the treatment of SCCs with a response rate of 10-25% [3,4]. Exploring key molecular factors that contribute to the immunosuppressive tumour microenvironment offers opportunities to identify new targets for cancer immunotherapy to treat SCCs [5,6].

IL1RL1 (also known as ST2) is the receptor for the alarmin protein interleukin-33 (IL-33) that is constitutively expressed and located in the nucleus of keratinocytes, endothelial cells, or stromal fibroblasts under normal conditions [7]. In response to damage-associated molecular signals, IL-33 is released from the nucleus through endogenous or exogenous protease cleavage and leads to activation of IL1RL1-expressing innate and adaptive immune cells. The IL-33/IL1RL1 signalling axis was originally discovered to trigger type 2 immunity via IL1RL1-expressing T helper 2 (Th2) cells [8], where it plays a pivotal role in various conditions, including tissue repair, allergy and cancer [9]. Administration of recombinant IL-33 protein has been shown to promote the expansion and function of FOXP3^+^ regulatory T cells (Tregs) in the intestine via directly acting on IL1RL1^+^FOXP3^+^ Tregs [10], or through stimulating CD11c^+^ dendritic cells (DCs) in the spleen [11], suggesting a tolerising role of IL-33 signalling.

Recently, IL-33/IL1RL1 signalling was shown to activate the TIR/MyD88/NF-κB pathway leading to the production of inflammatory cytokines/chemokines and contributing to the progression of lung cancer [12,13], gastric cancer [14], colorectal cancer [15] and HRAS-driven SCC [6]. Mechanistically, the IL-33/IL1RL1 signalling pathway enhanced the infiltration of immunosuppressive immune cells (such as type 2 innate lymphoid cell (ILC2), IL1RL1^+^ Tregs and IL1RL1^+^FcɛRIα^+^ macrophages, thereby creating a tumour-promoting environment. Conversely, the IL33/IL1RL1 axis also enabled anti-tumour immunity in some other cancer models, such as melanoma [16,17] and colorectal cancer [18], by enriching effector CD8^+^ T cells within the tumour. The immunoregulatory functions of the IL33/IL1RL1 axis are hence highly dependent on the context and the cancer models.

Here, we show that the IL-33/IL1RL1 axis plays a critical role in mediating Treg accumulation and immune suppression in a precancerous model of SCC. We identified that a subset of hyperproliferative keratinocytes contains increased levels of IL-33 within the cytoplasm, and this was associated with an increase in IL1RL1-expressing Tregs. We further show that systemic genetic deletion of IL1RL1 overcomes immune tolerance in HPV16-E7 oncoprotein-mediated epithelial hyperplasia, resulting in the partial rejection of neoantigen-expressing hyperplastic skin grafts. These results indicate that blocking the IL-33/IL1RL1 axis may be beneficial to restore effector immune responses in SCC.

## 2 Results

### 2.1 *Il33*-*Il1rl1* gene co-localisation occurs in both murine and human HPV^+^ intraepithelial neoplastic epithelium

A transgenic mouse model expressing the HPV16 E7 oncoprotein under control of the keratin 14 promotor (K14E7) presents with hyperproliferative dysplastic epithelium and is valuable to decipher mechanisms of immune suppression in the early stages of SCC development. We have previously shown that the murine K14E7 skin shares a high similarity in gene signatures with high-grade cervical intraepithelial neoplasia (CIN) in humans [19]. The K14E7 epithelium is infiltrated with clonally diverse *Il1rl1*^+^ Tregs [20], and Treg accumulation is associated with the inability of skin-resident antigen-presenting cells to prime effector T cell responses [[21], [22], [23]].

**Fig. 1:**
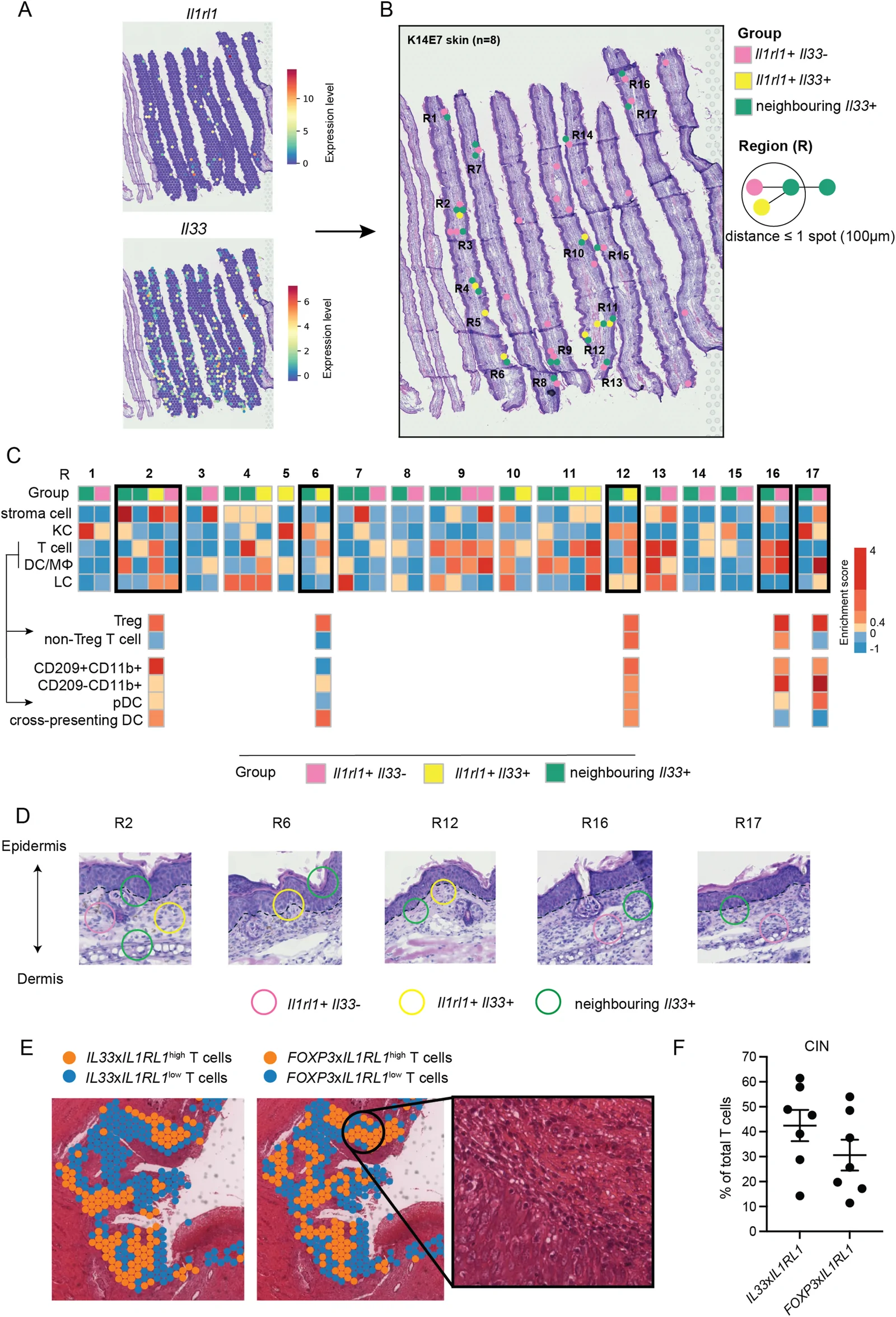
**Spatial transcriptomics predicts diverse sub-anatomical sites of *Il33 and Il1rl1* co-localisation in hyperplastic skin.** Spatial transcriptomic profiles of 8 longitudinal sections of K14E7 ear pinnae skin were generated. **(A)** Expression levels of *Il1rl1* (ST2) and *Il33* gene in each tissue-covered spot were visualized. **(B)** Regions (R1-R17) of *Il33*-*Il1rl1* co-localisation were identified by selecting and grouping neighbouring *Il1rl1*+ and *Il33*+ spots (spots that are within 100 μm, which is the distance between the centres of two neighbouring spots). Pink: Spots that express *Il1rl1* but no *Il33*; Yellow: Spots that express both *Il1rl1* and *Il33*; Green: Spots that express *Il33* and are adjacent to at least one *Il1rl1*+ spot. **(C)** Gene set enrichment analysis was performed to investigate cellular components within each spot in each *Il33*-*Il1rl1* co-localisation region. Enrichment scores of cell-type specific gene sets (stroma cells; keratinocytes; T cells; DC/Macrophages and LC) for each spot were visualized in a heatmap. Five representative *Il33*-*Il1rl1* regions (R2, R6, R12, R16, R17) were identified, and their enrichment scores of cell-subtype specific gene sets (T cell: Treg and non-Treg; DC/Macrophage: CD209^+^CD11b^+^, CD209^−^CD11b^+^, pDC, cross-presenting DC) were visualized. **(D)** H&E images of whole skin tissue were overlaid with *Il33*-*Il1rl1* co-localisation regions. **(E)** H&E images of a representative CIN sample were overlaid with transcriptomic Visium spots co-expressing either *IL33* and *IL1RL1* or *FOXP3* and *IL1RL1*. **(F)** Dot plot showing percentages of transcriptomic Visium spots co-expressing either *IL33* and *IL1RL1* or *FOXP3* and *IL1RL1* across 7 CIN samples.

To unravel the spatial relationship between IL1RL1-and IL-33-expressing cells in K14E7 skin and identify their cell types of origin, we applied spatial transcriptomic profiling of K14E7 skin (n = 8) using the 10X Genomics Visium platform. Tissue spots that expressed *Il33* and (or) *Il1rl1* transcripts were annotated into three groups (Fig. 1A and B): (1) *Il1rl1*^+^ spots (pink) expressed *Il1rl1* but not *Il33*; (2) *Il33*^+^ *Il1rl1*^+^ spots (yellow) expressed both *Il1rl1* and *Il33*; (3) neighbouring *Il33*^+^ spots (green) expressed only *Il33* but were located within 100 μm distance to their closest *Il1rl1*-expressing spots (i.e. close to either *Il1rl1*^+^ spots or *Il33*^+^ *Il1rl1*^+^ spots). Although one might expect similar numbers of *Il33*^+^ and *Il1rl1*^+^ spots if IL-33 drives IL1RL1^+^ cells, the lower number of IL1RL1 dots likely reflects technical limitations of Visium, which has reduced sensitivity for transcripts in rare cell populations. This is likely compounded by the epithelial dominance of each Visium spot, which can mask signals from less abundant immune cell transcripts such as IL1RL1.

Regions of *Il33* and *Il1rl1* co-localisation were defined as *Il33*^+^ *Il1rl1*^+^ spots (yellow) or the regions where the neighbouring *Il33*^+^ spots were adjacent to *Il1rl1*-expressing spots (green + yellow/pink). These regions could exist as standalone structures (e.g., a single yellow spot or one green + yellow/pink combination) or merge together if they were located within 100 μm of other pink, green, or yellow spots. Conversely, isolated pink or green spots located more than 100 μm away from other pink, green, or yellow spots were not classified as regions of *Il33* and *Il1rl1* co-localisation. Using these criteria, a total of 17 *Il33*-*Il1rl1* co-localisation regions (R1-R17) were identified across 8 samples of K14E7 skin. As each Visium spot captures the transcriptome of approximately 1-10 cells [24]*,* we examined the cellular content of each co-localisation region via gene set activity analysis (AUCell package (V1.14)) using gene sets characteristic to stromal cells, keratinocytes, T cells, DC/macrophages and LCs (Fig. 1C). Most regions were enriched for heterogeneous cell types, implying potential cell-cell interactions. Given the prior knowledge that the IL-33/IL1RL1 signalling pathway underlies the crosstalk between KC/stromal cells and immune cells, we next focused on 5 regions (R2, R6, R12, R16, R17) that were enriched in gene signatures associated with both KC/stromal cells and immune cells. Specifically, these 5 selected regions exhibited high enrichment (scaled AUCell enrichment score > 0.4) for either KC or stromal cell signatures in green/yellow spots, together with high enrichment (scaled AUCell enrichment scores > 0.4) for at least one immune cell signature (T cells, DC/macrophages, or LCs) in pink/yellow spots. A secondary gene set activity analysis using immune cell subset signatures indicated that these 5 regions were all enriched for a Treg signature and at least one myeloid cell subtype signature. An overlay of the co-localisation regions with H&E images revealed that *Il33*-*Il1rl1* co-localisation was not restricted to specific skin layers and occurred in both dermal and epidermal layers of K14E7 skin (Fig. 1D).

To confirm the spatial association between T cells and *IL1RL1*/*IL33* co-expression in human tissues of high-grade cervical intraepithelial neoplasia (CIN), we analysed spatial transcriptomic profiling on samples derived from 7 CIN3 patients [25]. We annotated tissue regions enriched for a T cell gene signature and further distinguished T cell-enriched spots co-expressing *IL33* and *IL1RL1* (orange) from those that did not (blue) (Fig. 1E). A significant percentage of T cell-enriched spots co-expressed *IL33* and *IL1RL1*, ranging from 14% to 61% across different CIN samples (Fig. 1F). These spots were widely distributed in regions adjacent to CIN neoplastic regions. Notably, a substantial percentage (11% to 54%) of *IL1RL1*-expressing spots co-expressed *FOXP3*, indicative of a Treg identity and consistent with observations in K14E7 skin. These *IL1RL1*^+^ *FOXP3*^+^ spots were frequently located in close spatial proximity to *IL33*-expressing regions.

### 2.2 *Il33*^+^ basal hyperproliferative keratinocytes co-express *Gpr15l* and MHCII-related genes

**Fig. 2:**
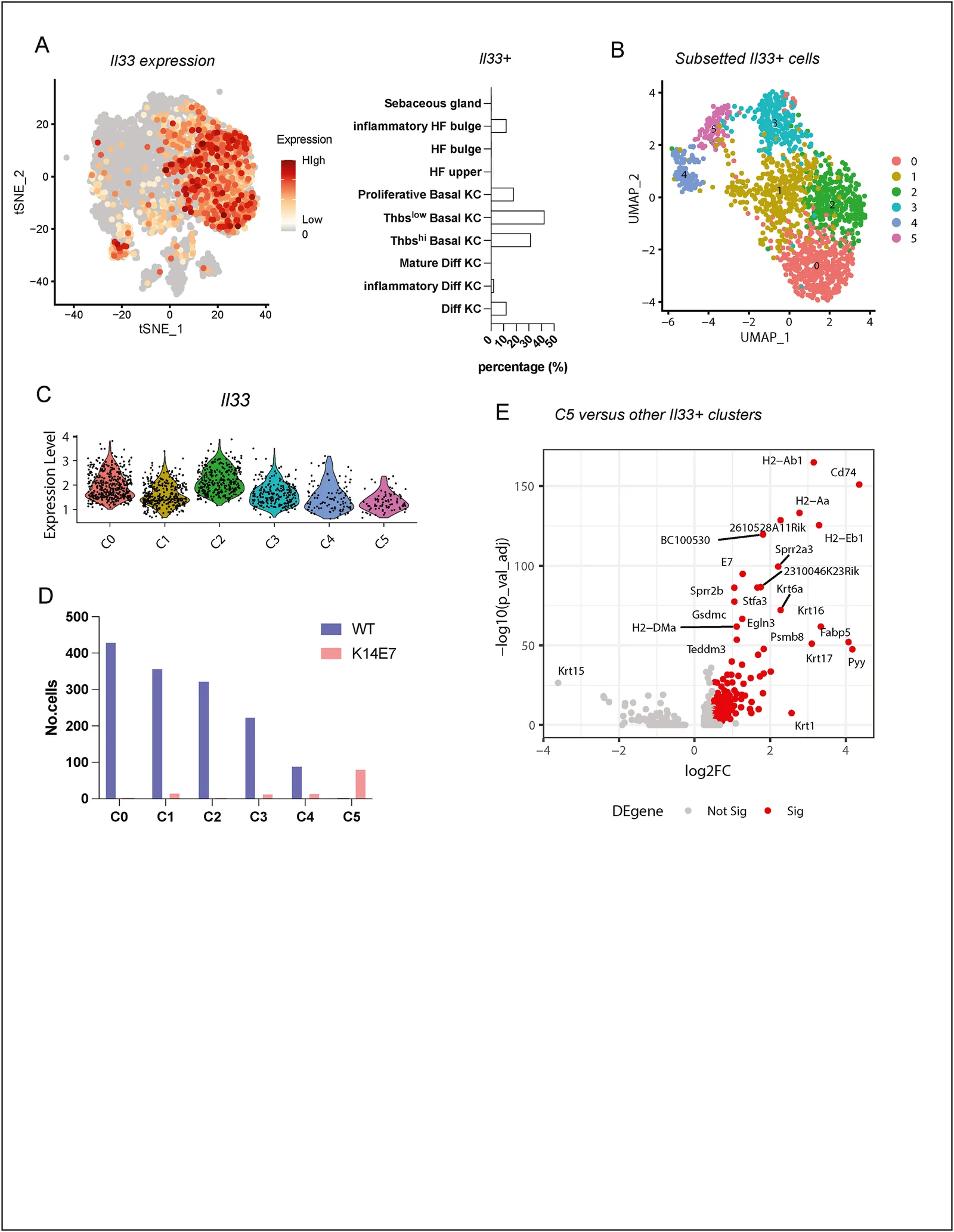
**Basal keratinocytes in hyperplastic skin co-express *Il33*, *Gpr15l* and MHCII-related genes.** The scRNAseq data of CD45^−^epithelial cells was derived from the published data from K14E7 and C57BL/6 ear pinnae skin [26] and was re-analysed for examination of *Il33* expression patterns. **(A)** Left: Expression levels of *Il33* across all epithelial cells were visualized. Right: The percentage of *Il33*^+^ cells (cells that have non-zero *Il33* expression) was calculated for each keratinocyte cell cluster. **(B)** All *Il33*^+^ keratinocytes from K14E7 and C57BL/6 epidermis were subsetted from the original dataset. Unsupervised Seurat clustering of these *Il33*^+^ cells resulted in six clusters (C0-C5). **(C)** Expression levels of *Il33* across these six clusters were compared. **(D)** The absolute number of K14E7 and C57BL/6-derived cells in each of these six clusters was summarised. **(E)** Volcano plot depicts differentially expressed genes (DEGs) (adj p < 0.001, Red) upregulated in C5 cells compared to C0-C4.

Skin hyperplasia induces a series of transcriptomic changes in keratinocytes, as indicated by our previously published transcriptomic and chromatin accessibility studies of keratinocytes from normoplastic and hyperplastic epidermis [26,27]. To investigate whether epidermal hyperplasia induces specific transcriptomic cell states in *Il33*^*+*^ keratinocytes, we re-analysed our published single-cell RNA-seq data for a detailed phenotyping of the *Il33*^*+*^ keratinocyte population in murine K14E7 epidermis. Based on the previously established keratinocyte cluster annotation [27], we revealed that *Il33* expression was largely restricted to basal keratinocytes in both normal and hyperplastic K14E7 skin (Fig. 2A). We isolated all keratinocytes expressing *Il33* (132 from K14E7 and 1429 from normal skin) and subjected these to further clustering, resulting in 6 transcriptionally diverse cell states (C0-C5) (Fig. 2B). *Il33* was highest expressed in clusters C0 and C2 and lowest expressed in cluster C5 (C0/C2 vs C5: p < 1e-20) (Fig. 2C). Notably, most *Il33*^+^ cells within clusters C0-C4 were derived from normal epithelium, while all *Il33*^+^ cells within C5 were K14E7-derived (Fig. 2D), demonstrating that *Il33* gene expression was reduced in K14E7 keratinocytes. This overall *Il33* transcript reduction in K14E7 skin was confirmed using quantitative reverse transcription PCR of bulk dermis and epidermis (Fig. S1). Using existing bulk chromatin accessibility sequencing data of keratinocytes from normal and K14E7 hyperplastic epidermis [28] revealed 4 differential enriched sites (DES) within the *Il33* gene loci, where two were more accessible (DES 1 and 4), and two were less accessible (DES 2 and 3) in K14E7 keratinocytes, suggesting transcriptional dysregulation of the *Il33* gene in hyperproliferative epithelial cells. Despite the overall reduced frequency of *Il33*-expressing cells in K14E7 skin, K14E7 keratinocytes exhibited unique phenotypes. We compared transcriptomic changes between the *Il33*^+^ K14E7-derived cells (C5) and all remaining *Il33*^+^ cells (C0-C4, primarily derived from normal skin). *Il33*^+^ K14E7 keratinocytes significantly upregulated genes including *E7*, *Cd74*, MHCII-related genes and the chemokine gene *2610528A11Rik* (also known as *Gpr15l*) (Fig. 2E). GPR15L, commonly expressed by epithelial cells and upregulated in chronic inflammatory skin lesions [29], is a ligand to GPR15 expressed by lymphocytes, and plays a role in the gut homing and homeostasis of Tregs [30]. Together with the expression of MHCII genes and CD74, this suggests that K14E7 basal keratinocytes have unique mechanisms to interact with skin-resident or skin-infiltrating T cells.

### 2.3 A subset of cells in hyperproliferative epithelium contains high levels of cytoplasmic IL-33

**Fig. 3:**
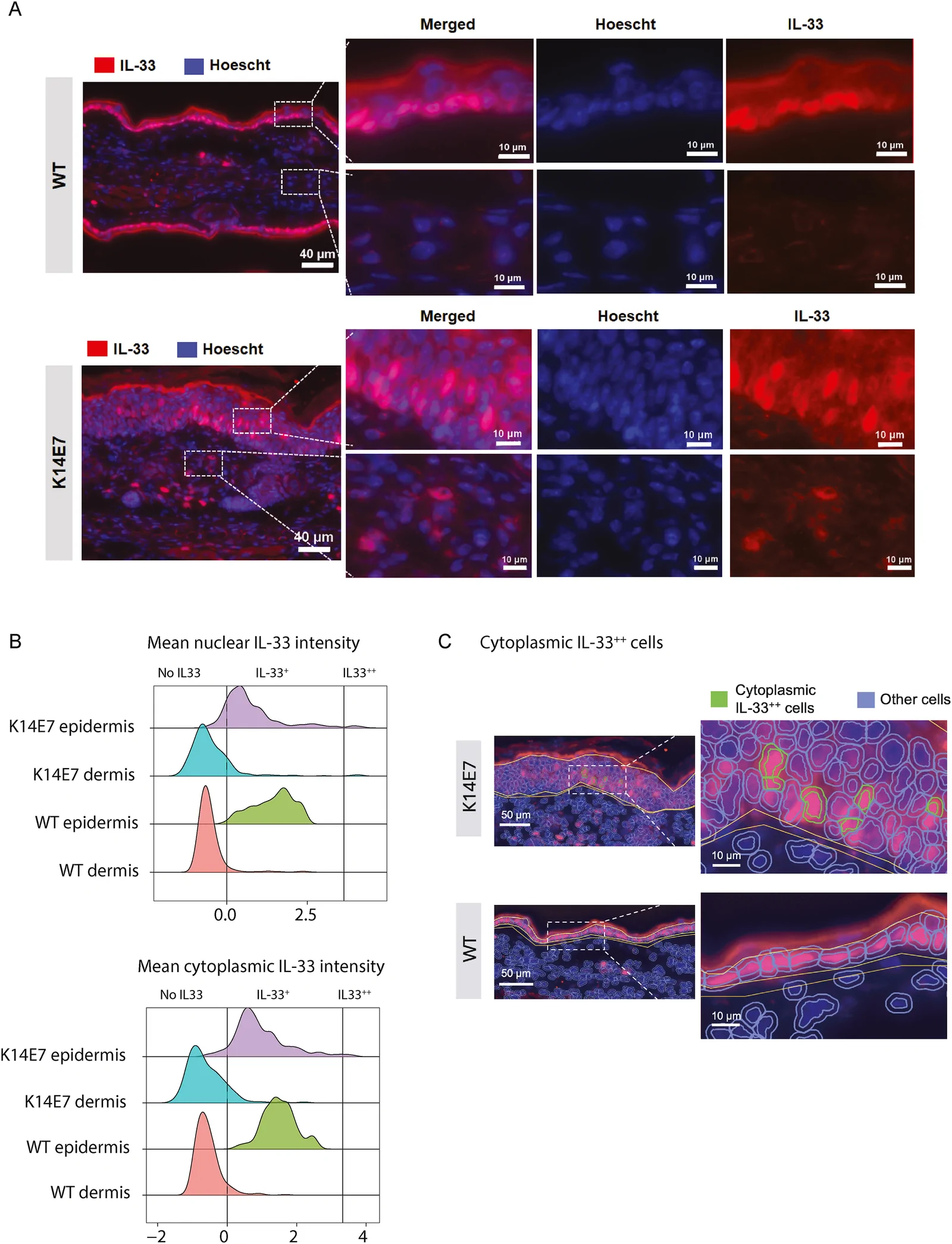
**A subset of cells in hyperproliferative epithelium contains high cytoplasmic IL-33 protein levels. (A)** Immunohistochemical analysis of IL-33 protein distribution (red fluorescence) with nuclear counterstaining (Hoescht, blue) was performed in ear pinnae skin of K14E7 and wild-type (WT) mice. **(B)** The mean intensity of nuclear and cytoplasmic IL-33 was determined after automated nucleus and cytoplasm segmentation. Intensity values were Z-normalized and categorized into three groups: No IL-33 (no detectable IL-33), IL-33^+^ (low to moderate intensity) and IL-33^++^ (high intensity). **(C)** Cells expressing high cytoplasmic IL-33 (Green: mean intensity larger than 3.5) were displayed on images.

During homeostasis, IL-33 is a nuclear cytokine and is released to the cytoplasm and cell surface in response to nonlethal mechanical stress [31]. We hence sought to determine the intracellular localisation of IL-33 protein in hyperproliferative K14E7 keratinocytes. Immunostaining of wildtype (WT) and K14E7 skin revealed that IL-33 was evenly distributed in the basal layer of the WT epidermis, and this pattern was disrupted in the K14E7 epidermis (Fig. 3A). Overall, and in line with the transcriptomic data, the mean intensity of both nuclear and cytoplasmic IL-33 was higher in WT epidermal cells compared to K14E7 (Fig. 3B). However, we identified a small proportion (2%) of cells in K14E7 epidermis with a high intensity of nuclear and cytoplasmic IL-33 (IL-33^++^), and which was absent in WT epidermis (Fig. 3B and C), suggesting that a subset of cells in the hyperproliferative epithelium with increased cytoplasmic IL-33 availability may communicate with IL1RL1^+^ adjacent cells.

### 2.4 Hyperplastic epithelium is enriched with *Il1rl1*^+^ myeloid and T cells

**Fig. 4:**
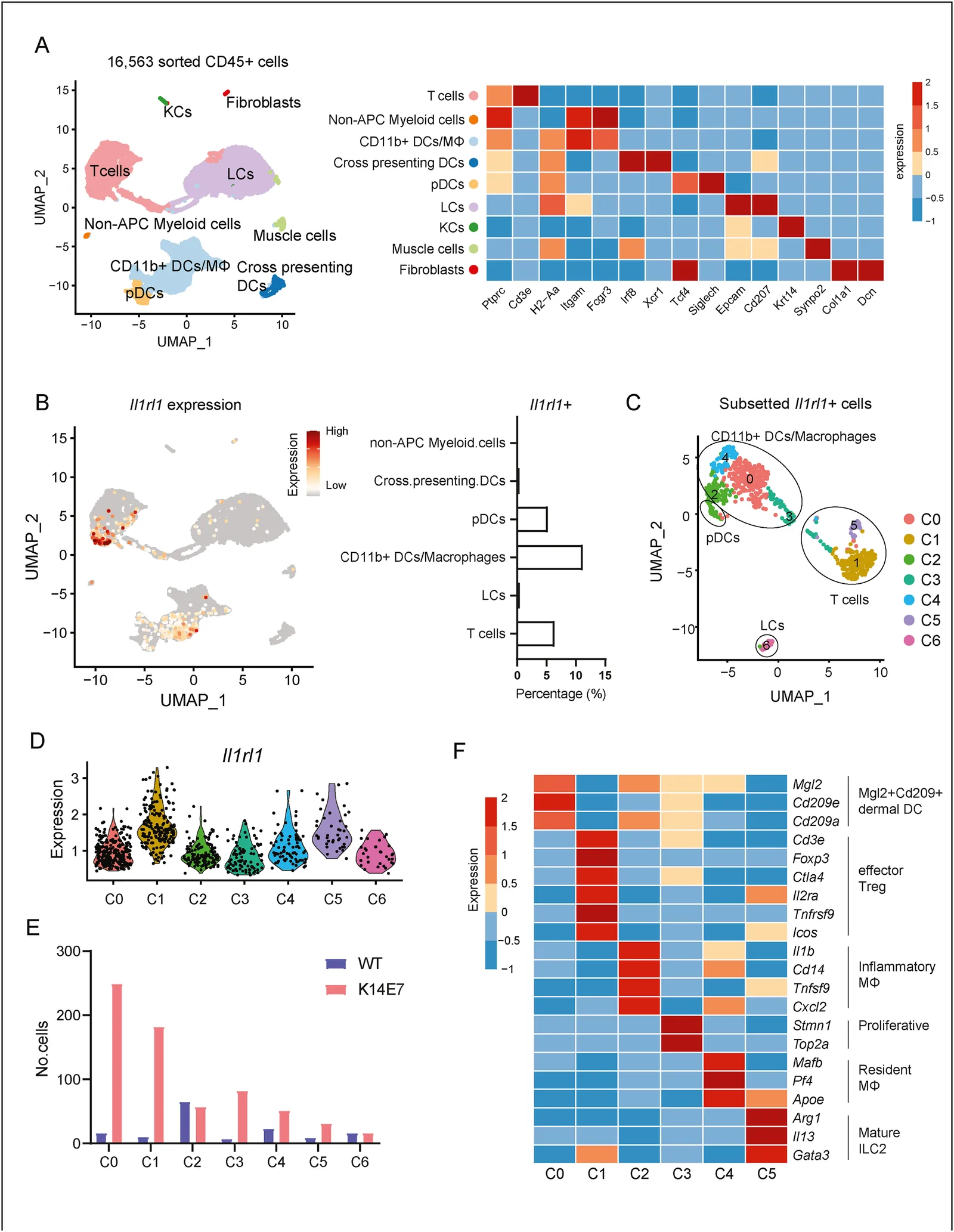
***Il1rl1* (ST2) gene is dominantly expressed by Foxp3^+^ effector Tregs and a subpopulation of CD11b** **^+^** **myeloid cells in hyperplastic skin.** The scRNAseq dataset of CD45^+^ cells from K14E7 and C57BL/6 ear pinnae-derived epidermis was derived from a previously published study [34] and was re-analysed for examination of *Il1rl1* (ST2) gene expression. **(A)** Unsupervised clustering of a total of 16563 CD45^+^ cells revealed 9 clusters. Mean expressions of representative genes specific for each of these clusters were visualized in a heatmap. **(B)** Left: UMAP plot depicts single-cell expression pattern of *Il1rl1* gene in each cell cluster. Right: For each cluster, percentage of cells that have non-zero *Il1rl1* expression was calculated. **(C)** CD45^+^ cells that have non-zero *Il1rl1* expression were subsetted. Unsupervised clustering resulted in seven subclusters (C0-C6). **(D)***Il1rl1* expression across these seven subclusters were compared. **(E)** The absolute number of K14E7 and WT epidermis-derived cells in each subcluster was summarised. **(F)** Top DEGs in each subcluster was visualized in a heatmap.

*Il1rl1* is highly expressed by a subpopulation of tissue-resident Tregs in K14E7 skin when compared to normal skin [20]. In this study, we observed significantly increased levels of *Il1rl1* in K14E7 epidermis (Fig. S1). As described above, K14E7 basal keratinocytes expressed a unique combination of *Il33*, *Gpr15l* and MHCII-related genes, suggesting that K14E7 skin may exhibit a preference in the recruitment of IL1RL1^+^ immune cells, especially IL1RL1^+^ Tregs, when compared to normal skin. To further investigate this, we reanalysed our previously published single-cell RNA-seq data of CD45^+^ immune cells from WT and K14E7 epidermis [32] for a detailed phenotyping of *Il1rl1*^+^ immune cell populations. Based on a basic cluster annotation for major cell types, we found that *Il1rl1* was highly expressed by a subset of T cells and was also expressed at relatively low levels by CD11b^+^ myeloid cells (Fig. 4A and B). However, we did not observe IL1RL1 protein expression on antigen-presenting cells in K14E7 epithelium (Fig. S2). After subsetting and re-clustering all *Il1rl1*^+^ cells, we identified 7 cell subclusters (C0-C6) (Fig. 4C). C1 and C5 represented T cells that expressed the highest levels of *Il1rl1* (Fig. 4C and D). C2 (pDCs) and C3 (myeloid and T cells) expressed the lowest levels of *Il1rl1* (Fig. 4C and D). C0 (CD11b + myeloid cells) and C1 (T cells) were almost exclusively represented by K14E7 cells and accounted for the majority of *Il1rl1*+ cells (Fig. 4E). Differential gene expression analysis revealed that the C0 CD11b + myeloid cells co-expressed *Mgl2* and *CD209* (Fig. 4F), suggesting these are dermal conventional type 2 DCs (DC2), while the C1 T cells expressed *Foxp3*, *Ctla4*, *Il2ra*, *Tnfrsf9* (4-1BB) and *Icos*, suggesting that these are effector Tregs [33]. Interestingly, K14E7 skin also showed an increase in C5 cells, which highly expressed ILC2-related molecules *Il13* and Arg*1*.

### 2.5 Genetic deletion of *Il1rl1* leads to a decrease in Tregs and an increase in cDC1 in hyperplastic epithelium

**Fig. 5:**
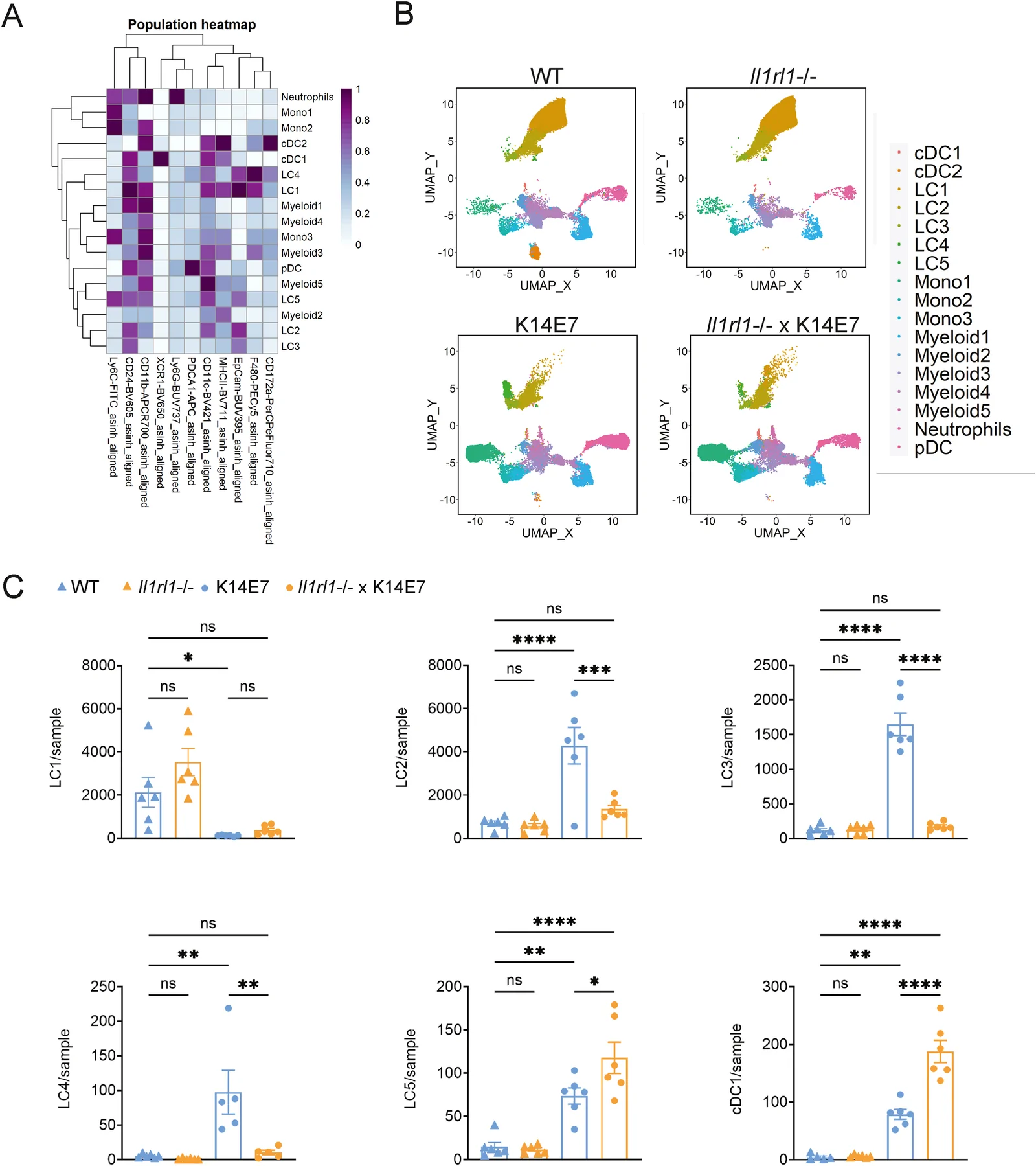
**Genetic deletion of *Il1rl1 (*ST2) leads to a reduction of LCs and an increase in cDC1 within the hyperplastic epithelium.** Single cell suspensions from ear pinnae-derived epidermis was analysed using flow cytometry. **(A)** A heatmap displays the expression levels of representative protein features specific to each cluster. **(B)** FlowSOM and UMAP analysis projects 17 clusters of myeloid cells derived from the epidermis of WT, *Il1rl1*^−/−^, K14E7 and *Il1rl1*^−/−^xK14E7 mice. **(C)** Absolute numbers of different subsets of LCs and cDC1 infiltrated in the epidermis of different experimental groups. Data are presented as mean ± SEM (n = 6) of two pooled independent experiments.

**Fig. 6:**
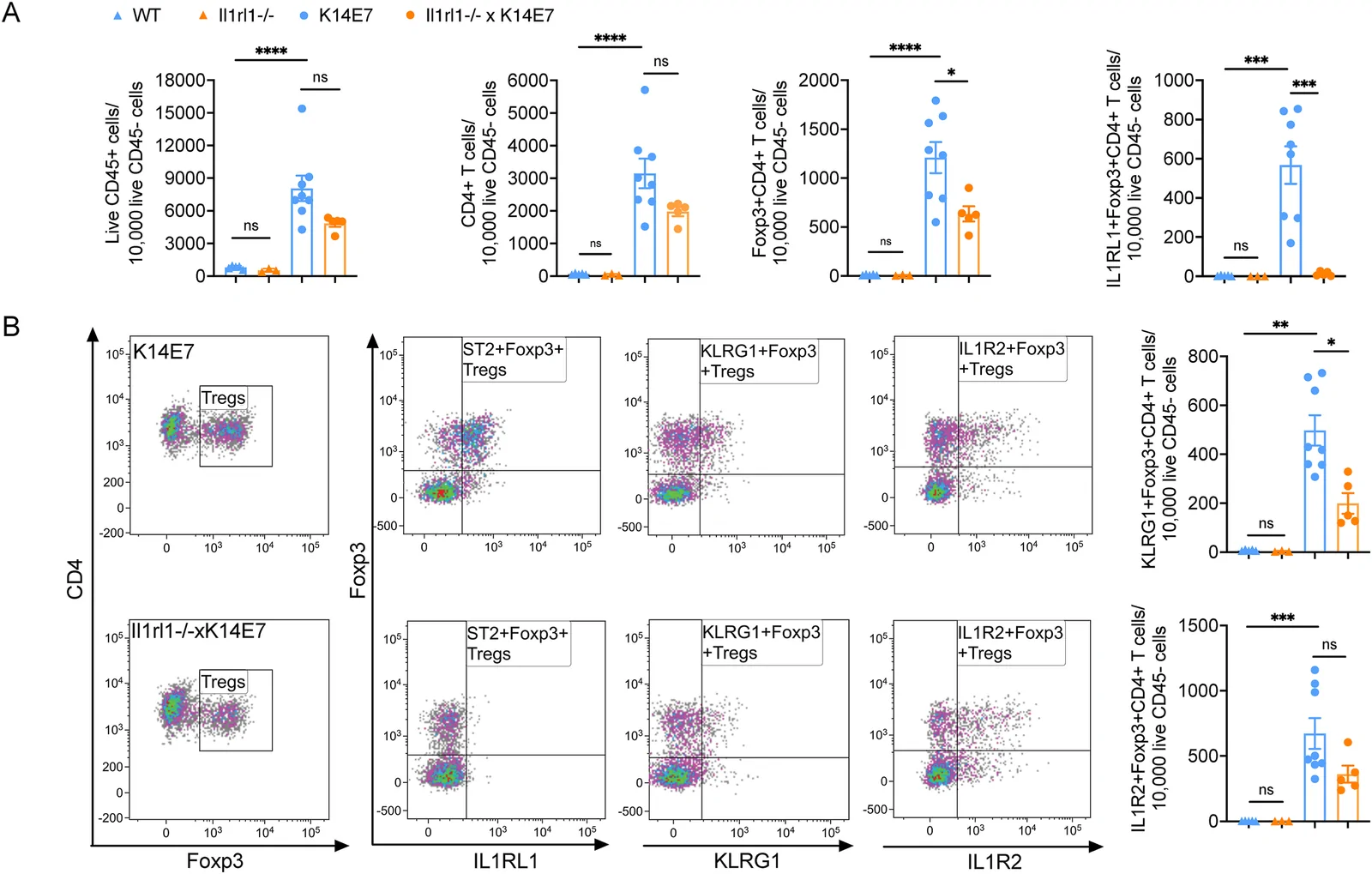
**Genetic deletion of *Il1rl1 (*ST2) results in a significant reduction of activated Tregs in hyperplastic skin.** The bar charts and dot plots of CD45^+^ immune infiltrates, CD4^+^ T cells, Tregs, and activated KLRG1^+^Tregs and IL1R2^+^ Tregs relative to keratinocytes derived from the ear pinnae skin of WT, *Il1rl1*^−/−^, K14E7 and *Il1rl1*^−/−^xK14E7 mice. Data are presented as mean ± SEM (n = 3-8) of pooled two independent experiments.

To investigate the functional relevance of the IL-33/IL1RL1 axis in K14E7 skin, we generated *Il1rl1* gene-depleted mice (*Il1rl1*^−/−^ and K14E7x*Il1rl1*^−/−^ mice). Of note, *Il1rl1* gene deletion led to obvious compositional changes of the K14E7 skin immune infiltrates, as indicated by comprehensive flow cytometric immunophenotyping of myeloid and T cells (Fig. 5, Fig. 6). Unsupervised clustering analysis of 11 myeloid cell-associated protein markers allowed the identification of 17 myeloid cell subtypes or states, which were grouped broadly into LCs, monocytes, neutrophils, cDC1, cDC2, pDCs and other myeloid cell states that did not clearly align with these lineages (Fig. 5A). The distribution of myeloid cell states varied between experimental groups (Fig. 5B). Notably, based on the differential expression of 11 myeloid markers, we identified 5 different LC states (LC1-LC5) across all samples. In line with our previous research [22,27,32] [23] showing that K14E7 epidermis lacked fully mature LCs, we found that the dominant LC state in WT (LC1) expressed high levels of EPCAM, CD11c, CD24, CD11b and MHCII, and this LC state was largely absent in K14E7 epidermis (Fig. 5C). In contrast, K14E7 epidermis was enriched with LC states (LC2, LC3 and LC4) that expressed low levels of MHCII and CD11b. Interestingly, the absence of *Il1rl1* led to a decrease in LC2, LC3 and LC4 cell states in K14E7 epidermis. However, the WT-resident LC1 cell state was not replenished after *Il1rl1* deletion in K14E7 skin (Fig. 5C). While hyperproliferative K14E7 epithelium contained an infiltrate of monocytes, the absence of *Il1rl1* had no impact to the numbers of monocytes (data not shown). Interestingly, the number of cross-presenting cDC1 was significantly increased in K14E7-*Il1rl1*^−/−^ skin compared to K14E7 (Fig. 5C).

While the number of total CD45^+^ immune cells and CD4^+^ T cells relative to keratinocytes was not different between K14E7-*Il1rl1*^−/−^ and K14E7 epidermis, the frequency of Tregs was significantly reduced in the absence of *Il1rl1* (Fig. 6A and B). This reduction was primarily attributed to the complete depletion of the IL1RL1^+^ Treg subpopulation, as well as a decrease in activated KLRG1^+^ Tregs and IL1R2^+^ Tregs (Fig. 6A and B). Interestingly, Tregs were reduced by approximately 50% in K14E7x*Il1rl1*^−/−^ epidermis compared to K14E7 yet were still significantly increased compared to normoplastic WT skin, suggesting that other mechanisms besides the IL-33/IL1RL1 signalling axis contribute to effector Treg accumulation in hyperplastic epithelium.

### 2.6 *Il1rl1* deletion leads to rejection of K14E7 skin grafts

Despite expression of the HPV16 E7 oncoprotein as neoantigen, K14E7 skin is tolerated when grafted to immunocompetent animals. We have previously identified contributing factors to K14E7 graft tolerance, such as natural killer T cells [35], mast cells [36], T cells [37] and the soluble mediators IFN-γ [38], IL-17 [39] and IDO [40]. However, the mechanisms that lead to the establishment of this highly complex tolerogenic immune environment remain largely unknown.

**Fig. 7:**
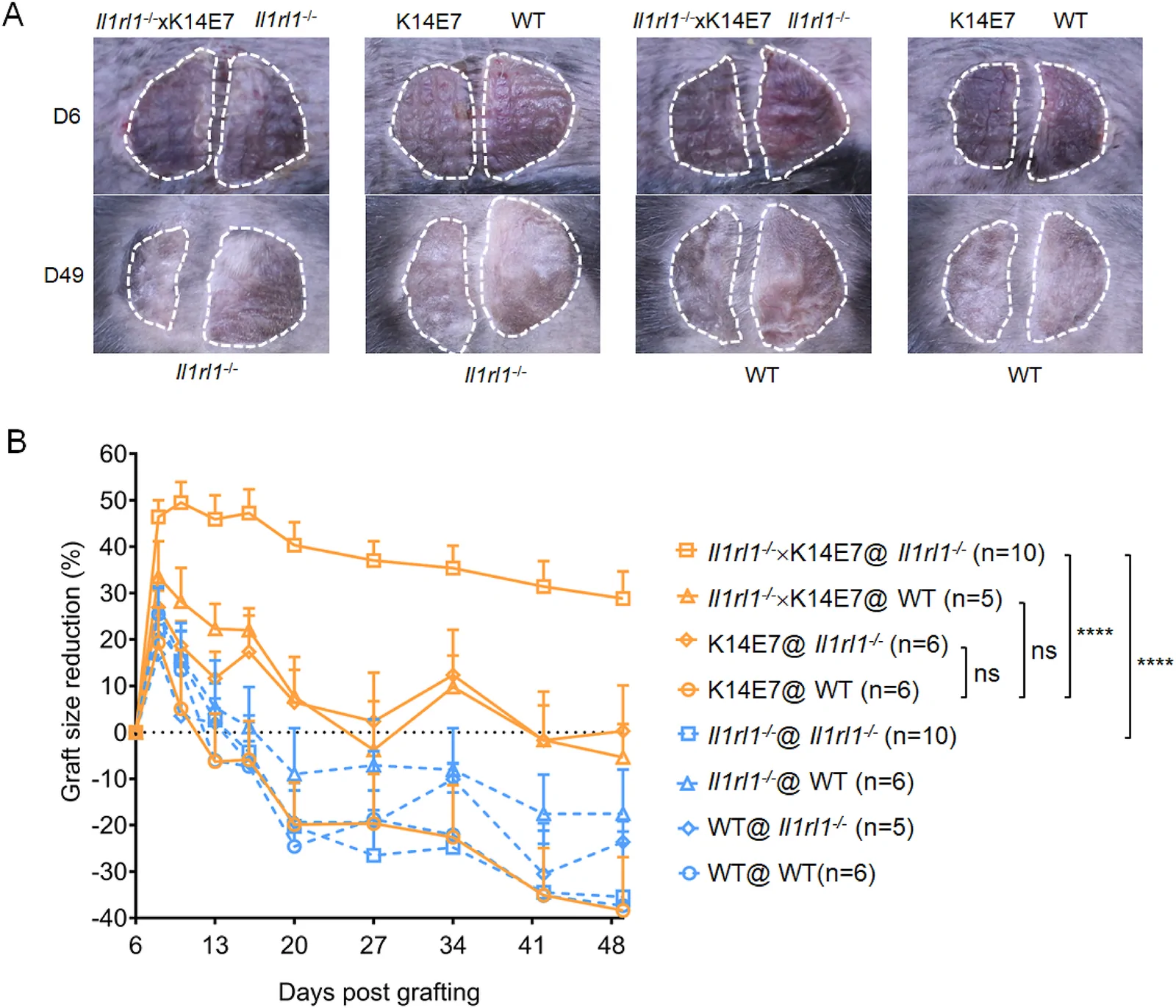
**Genetic deletion of *Il1rl1 (*ST2) overcomes the immune tolerance of K14E7 skin grafts.** Ear pinnae skin of WT, *Il1rl1*^−/−^, K14E7 and *Il1rl1*^−/−^xK14E7 mice was grafted to flanks of WT and *Il1rl1*^−/−^ mice and observed for graft tolerance and rejection. **(A)** Representative photographs of skin grafts on days 6 and 49 after skin transplantation. **(B)** The percentages of skin graft reduction over time were calculated from the photographs with a ruler and using Fiji ImageJ software (n = 5-10). Data are presented as mean ± SEM and are pooled from two independent experiments.

To evaluate if IL-33/IL1RL1 signalling contributes to K14E7 skin graft tolerance, we transplanted skin of *Il1rl1*^−/−^xK14E7 mice and controls to WT and *Il1rl1*^−/−^ animals, respectively. Only *Il1rl1*^−/−^xK14E7 skin grafts transplanted to *Il1rl1*^−/−^ recipient animals resulted in a significant reduction of skin graft sizes, while *Il1rl1*^−/−^xK14E7 skin grafts transplanted to *Il1rl1*-sufficient recipients remained tolerated (Fig. 7A and B). This demonstrates that immune cells of *Il1rl1-*sufficient recipient animals can migrate into *Il1rl1*^−/−^xK14E7 skin grafts to compensate for the loss of *Il1rl1* in the grafts and maintain immune tolerance. However, when *Il1rl1* is absent from both skin graft and the graft recipient, K14E7 immune tolerance can be partially overcome, leading to skin graft shrinkage.

## 3 Discussion

Intraepithelial neoplasia is a precursor lesion of many SCC types, including HPV-mediated and HPV-independent SCCs of the head and neck, anogenital squamous epithelia (e.g. cervical, vaginal, vulvar, penile and anal), and cutaneous UV-mediated SCCs. The K14E7 murine model of HPV16 E7 oncoprotein-mediated epithelial hyperplasia mimics both cutaneous and mucosal intraepithelial neoplastic stages [19,41]. Our previous research has determined immune modulatory mechanisms present in K14E7 epithelial hyperplasia that are characterised by alterations in skin-resident LCs and an accumulation of Tregs [22,23,27,32]. However, the mechanisms that lead to Treg accumulation remained unknown. In this study, we demonstrate a role for keratinocyte-derived IL33 and Treg-derived IL1RL1 expression in Treg recruitment and immune suppression.

LCs are considered the main professional antigen-presenting cells in the epidermis. They directly interact with epidermis-resident memory and regulatory T cells during steady state and environmental insults to maintain tolerance and facilitate tissue repair [42]. We have previously demonstrated that LCs in K14E7 skin display reduced expression of MHCII [22], have limited capability to take up and process antigen [23], and display a semi-mature/inhibitory transcriptomic profile [32]. We have further demonstrated that this murine K14E7-drived LC transcriptomic gene signature is enriched in transcriptomic data of human cervical cancer samples [32]. This is in line with recent research demonstrating that UVB radiation also leads to a downregulation of MHCII in LCs in human skin [42]. Furthermore, the K14E7 epidermis has an accumulation of clonally diverse tissue-resident Tregs expressing either KLRG1 or IL1R2 [20]. Tregs are known to express IL1RL1, the receptor for the alarmin cytokine IL33. We hence questioned whether the IL-33/IL1RL1 signalling axis may play a role in Treg recruitment and immune suppression.

This study identified a distinct heterogeneity in IL1RL1-expressing immune cells. While transcripts of IL1RL1 were expressed by Tregs, pDCs, cDC2, and macrophages in K14E7 skin, we could only confirm protein expression on Tregs. IL1RL1^+^ Tregs further expressed *Foxp3, Ctla4, Il2ra, Tnfrsf9* (4-1BB) and *Icos,* which are critical molecules in suppressing excessive immune responses. Numerous inflammatory and anti-inflammatory roles of IL-33 on immune cells have been described, including both activation and polarisation towards regulatory functions in both myeloid and lymphoid immunocytes [43]. However, we did not observe IL1RL1 expression on CD8^+^ T cells or NK cells, cell types that have been implicated in carrying out IL-33-mediated anti-tumour activities [20], suggesting that IL-33 may play a tumorigenic role in the K14E7 skin.

Interestingly, while the overall IL-33 chromatin accessibility was dysregulated and the IL-33 gene transcript was reduced in K14E7 skin, cell type analysis revealed that IL-33 in K14E7 skin was expressed by a unique basal keratinocyte cell state, which also expressed MHCII, suggesting that these cells may be able to directly interact with CD4^+^ T cells. Additionally, imaging revealed that a subset of epithelial cells in K14E7 epidermis expressed high levels of cytoplasmic IL-33, suggesting increased bioavailability of IL-33 in a proportion of K14E7 keratinocytes. Hence, the overall reduced frequency of IL-33 transcript-expressing cells in K14E7-resident keratinocytes may not be relevant to its biological role in immune cell recruitment.

The chronic inflammatory and hyperproliferative state of K14E7 keratinocytes alters cellular stress responses and may disrupt nuclear retention signals, leading to IL-33 cytoplasmic release. Indeed, previous research has demonstrated that the hyperproliferative K14E7 skin exhibits a transcriptomic signature of a cytoskeletal response to injury, defined by chronic expression of stress keratins, a response that is also apparent in human actinic keratosis lesions, a pre-stage to cutaneous SCC [44]. Taniguchi et al. demonstrated that antioxidant activity in squamous carcinoma cells led to the cytoplasmic release of IL-33 through the transcription factor NRF2, and induced TGF-b in IL-33-responding macrophages [45]. While they have demonstrated a paracrine IL-33-TGF-b feedforward loop, TGF-b is also an important factor in the differentiation of Tregs.

After protease cleavage of the nuclear domain, the mature cytoplasmic and extracellularly released IL-33 has a 10-30 times higher biological activity by interacting with specific receptors on immune cells [46] [47]. Hence, the consideration of protein location of IL-33 is as important as transcript expression levels. Moreover, the presence of cytoplasmic IL-33 in certain dermal cells of K14E7 skin indicates that the stress response extends beyond the hyperproliferative epidermis, potentially establishing a broader immune-suppressive network that enables recruitment of IL1RL1-expressing cells. These immunofluorescence findings are consistent with the gene-level observations from our spatial transcriptomic analysis, further supporting the role of IL-33/IL1RL1 interactions in immune modulation in the hyperproliferative epithelium.

The cytoplasmic enrichment of IL-33 in a proportion of K14E7 keratinocytes may represent a key mechanism by which the hyperplastic epithelium influences the local immune landscape, by promoting Treg accumulation and impairing effector T cell responses. The notion that a population of K14E7-derived basal epithelial cells co-expressing E7, IL-33, stress response keratins and genes of the MHCII antigen presentation pathway further suggests that these cells may directly interact with CD4^+^ T cells and Tregs. The co-localisation of *IL33* and *IL1RL1* was further observed in human CIN tissues. Indeed, genetic deletion of IL1RL1 led to a reduction in Tregs in K14E7 skin. Further, using a functional model of skin grafting, this study demonstrates a significant increase in skin graft rejection after genetic deletion of IL1RL1 in the graft and the host. Importantly, IL1RL1-sufficient hosts retained tolerance towards K14E7 skin grafts, suggesting that IL1RL1-sufficient immune cells from graft hosts can migrate into K14E7-*Il1rl1*^−/−^ skin grafts and establish immune tolerance. It underscores the critical importance of a systemic IL1RL1-targeting strategy to overcome immune tolerance.

A limitation of this study is that it examined mechanisms of HPV16 E7-mediated immune modulation in murine cutaneous epidermis, whereas HPV16 physiologically infects human mucosal epithelium, including the cervix and oropharynx, and where the normal immune composition differs from that of murine cutaneous skin. This limitation was partly overcome by validating observations made in murine skin using available human datasets of cervical neoplasia. Future studies may aim to use mucosal intraepithelial neoplastic models and cell-type-specific IL1RL1 deletion as well as IL-33 deletion models to establish specific contributions of IL1RL1-expressing Tregs and other immune cells to this functional immune-regulatory IL33-IL1RL1 signalling axis in SCC. Furthermore, the clinical relevance of the IL-33/IL1RL1 axis should be assessed by examining comparable cell-cell crosstalk mechanisms in both HPV-dependent and HPV-independent SCCs.

Our data highlight the promise of targeting the IL-33/IL1RL1 axis, potentially to interrupt the interaction between IL33-expressing hyperproliferative keratinocytes and IL1RL1^+^ Tregs, thereby reversing immune suppression and boosting anti-tumor responses in SCC. A recent first-in-human phase 1 study of a IL1RL1-targeting monoclonal antibody demonstrated safety and tolerance [48]. However, due to its pleiotropic and context-dependent functions, systemic targeting of the IL-33/IL1RL1 axis may lead to undesired effects, supporting the rationale for considering localised delivery approaches such as topical application. Future studies should explore the therapeutic potential of blocking the IL-33/IL1RL1 axis in SCCs using antibodies, small molecules or natural compounds that have demonstrated promising preclinical outcomes in asthma and inflammatory diseases [49].

## 4 Methods

### 4.1 Mice

C57BL/6J (WT), *Il1rl1*^−/−^, K14E7 and *Il1rl1*^−/−^xK14E7 mice were bred and housed under specific pathogen-free conditions at the University of Queensland Biological Research Facility (UQ-BRF) located at the Translational Research Institute (TRI). C57BL/6J mice were purchased from the Animal Resources Centre (Perth, Australia). K14E7 mice were originally provided by P. Lambert, University of Wisconsin-Madison [50]. Six to twelve-week-old female mice were used in experiments. All animal experimental procedures were approved by The University of Queensland Animal Ethics Committee (2021/AE000460).

### 4.2 Preparation of single-cell suspensions

The ear pinnae skin samples were harvested from sacrificed mice, separated into dorsal and ventral halves and then chopped into small pieces using scissors in digestion buffer (DMEM medium (Gibco #41965) containing 2% heat inactivated FBS, 1 mg/mL Collagenase D (Roche, Basel, Switzerland) and 20 μg/mL DNAse I (Roche, Basel, Switzerland), 5 ml per a pair of ears). Following digestion under shaking (180 rpm) at 37 °C for 1h, the samples were passed through 70 μm strainers and then washed with FACS buffer to obtain the single cell suspensions.

Epidermal single-cell suspensions were prepared as previously described [27]. Briefly, after separation of dorsal and ventral ear pinnae, tissues were floated epidermis down in a solution containing Dispase II (Roche, Basel, Switzerland) for 1h. The epidermis was gently separated from the dermis using forceps, and further digested with Collagenase D and DNAse I for 30 min, followed by passing cells through a cell strainer.

To prepare splenocytes, the spleens were meshed through the 70 μm strainers and flushed with FACS buffer. The splenocytes were obtained after lysing red blood cells using ACK buffer.

### 4.3 Flow cytometry analysis

Single cells were incubated with antibodies for CD16/CD32 and viability stain. Myeloid cell flow cytometry was performed according to a previously optimised protocol using the published antibody clones and concentrations [51]. The T cell flow cytometry used the following antibody panel: viability stain (Live/Dead Fixable Aqua Dead Cell Stain Kit, ThermoFisher) and Fc block (anti-mouse CD16/CD32 antibody), PE-Dazzle-anti-mouse CD45 antibody (Clone 30-F11, BioLegend), PE-Cy7-*anti*-mouse CD25 antibody (Clone PC61, BioLegend), FITC-anti-mouse TCR-β antibody (Clone H57-597, Biolegend), AF700-*anti*-mouse CD4 antibody (Clone GK1.5, BioLegend), APC-Cy7-*anti*-mouse KLRG1 antibody (Clone 2F1, BioLegend), eFluor450-*anti*-mouse ST2 (IL1RL1) antibody (Clone RMST2-2, ThermoFisher Scientific), AF647-*anti*-mouse Foxp3 antibody (Clone MF-14, BioLegend), and PE-anti-mouse IL1R2 antibody (Clone 4E2, BD BioSciences).

Samples were acquired using the FACSymphony A5 flow cytometer (BD, USA), and analysed using FlowJo software (BD, USA), R programming and the Spectre software package for dimensionality reduction [52].

### 4.4 Immunofluorescence (IF) staining

Whole ear pinnae isolated from 8-week-old C57BL/6 and K14E7 mice were frozen, embedded in optimal cutting temperature (OCT) compound and cryosectioned at 7 μm thickness. Sections were rinsed with 1x PBS to soften the OCT material, fixed in ice cold acetone at room temperature for 30 min and washed in 1x PBS to remove excess acetone. Skin tissues were permeablized with 0.1% Triton X-100 in PBS (TX-100/PBS) for 10 min and washed with 0.025% TX-100/PBS twice for 5 min each. Blocking was performed for 1 h at room temperature with buffer containing 1% bovine serum albumin (BSA) and 10% normal donkey serum. The slides were incubated overnight at 4 °C with primary antibodies diluted in 1% BSA and washed with 0.025% TX-100/PBS. Sections were then incubated in the dark with secondary antibodies diluted in 1% BSA at room temperature for 1 h before mounting with ProLong Gold anti-fade mounting reagent. Primary antibodies used for staining includes goat IgG isotype control (R&D Systems RDSAB108C, 1:200) and goat anti-IL-33 (R&D Systems AF3626,1:200). Secondary antibodies used was donkey anti-goat, conjugated with Alexa Fluor 594 (Jackson Immunoresearch 705-585-147, 1:200). The slides were then rinsed with 0.025% PBST 3 times, and then counterstained with Hoescht (1:500) diluted in PBS for 15-20 min. Afterwards, the slides were then rinsed with PBS and then mounted with Prolong Gold Anti-fade mounting medium. The fluorescence images of skin section slides were taken by confocal microscopy (FV1200, Olympus, Tokyo, Japan).

### 4.5 Immunofluorescence (IF) image processing

Multi-channel fluorescence images were analysed using QuPath (version 0.5.1). The channel corresponding to nuclear staining was used to perform automated nucleus detection. To assess IL-33 intensity in the cytoplasm, cytoplasmic regions were defined as a 2 μm perinuclear zone surrounding each detected nucleus. Epidermal and dermal compartments were manually delineated, and IL-33 intensity was quantified separately within each compartment. For each image, the mean IL-33 intensity in each nuclear and cytoplasmic region was calculated and normalized by Z-score transformation. Based on these Z-scores, cells were categorized into three groups representing increasing levels of IL-33 expression: IL-33^-^ (no detectable IL-33), IL-33^+^ (low to moderate intensity), and IL-33^++^ (high intensity).

### 4.6 Spatial transcriptomics data generation

Fresh-frozen mouse ear skin samples were cryo-sectioned at 7 μm thickness, placed onto the designated capture area (6.5 mm × 6.5 mm) of the Visium slides. Tissues sections were permeabilised for 12 min, followed by construction of spatially tagged cDNA libraries using the 10x Genomics Visium Spatial Gene Expression 3’ Library Construction V1 Kit. Libraries were sequenced on NovaSeq 6000 (Illumina) with a minimum 50,000 read pairs per tissue covered spot.

### 4.7 Processing of spatial transcriptomics data

The Illumina sequencing output was processed using the 10x Genomics Space Ranger Software (v1.3.0). The command “spaceranger mkfastq” was used to demultiplex raw binary base call sequence files and generate FASTQ files. The command “spaceranger count” was used to align RNA-Seq reads to the pre-built mouse reference genome mm10 and to filter out low quality cell barcodes and unique molecular identifiers (UMI). Each spot was also aligned to the corresponding capture area image. The resulting single-spot gene count matrices were further processed using the Seurat package (V4.1.1) [53] and the STUtility package (V0.1.0) [54]. We filtered out transcripts that were present in fewer than 3 spots in each array. We removed spot with fewer than 200 transcripts. Cell-cell gene expression variation was normalized using the "LogNormalize" method with a scaling factor of 10,000 as implemented in the Seurat pipeline.

Gene set activity analysis was performed using the AUCell package (V1.14) [55]. AUCell calculates the “Area Under the Receiving Operator Curve” (AUC) score for each Visium spot by evaluating whether the input gene signature is enriched within the top-ranking genes (ordered by their expression values) for each spot. Gene sets corresponding to keratinocytes, stroma cells and immune cells (and their subtypes) were derived from public scRNA-seq datasets of K14E7 skin [26] [27] [32].

### 4.8 Reanalysis of published scRNA-seq data of murine K14E7 skin

Previously acquired scRNA-seq data of sorted KCs from WT and K14E7 mice were downloaded from https://www.ebi.ac.uk/arrayexpress/experiments/E-MTAB-6429/. Previously acquired scRNA-seq data of sorted CD45^+^ cells from WT and K14E7 mice were downloaded from https://www.ebi.ac.uk/arrayexpress/experiments/E-MTAB-8199/. Data were processed using the standard Seurat pipeline (V4.1.1).

### 4.9 Skin grafting

Skin grafting experiments were performed as our previously reported method [56]. Briefly, the ear skins were harvested from the sacrificed donor mice of C57BL/6J (WT), *Il1rl1*^−/−^, K14E7 and *Il1rl1*^−/−^xK14E7 mice, followed by separation into ventral and dorsal halves using forceps. The recipient WT and *Il1rl1*^−/−^ mice were grafted with dorsal ear skin on the flank region. The grafts were covered using Bactigras (Smith + Nephew) and dressing lengths (Elastoplast), followed by wrapping with bandages of Flex-wrap (Lyppard) and micropore tape. The bandages were removed at day 6 post grafting. The size changes of skin grafts were recorded weekly by taking digital photos together with a ruler, and quantified by using Fuji Imaging software.

### 4.10 Spatial colocalization analysis of human CIN3 samples

To investigate the spatial relationship between T cells and gene co-expression in human high-grade CIN samples, we performed a multi-step analysis of an established spatial transcriptomic dataset of 7 formalin‐fixed, paraffin‐embedded (FFPE) CIN3 samples [25] using the stlearn Python package [57]. First, a T cell signature was defined for each spatial spot by calculating a module score. This score was based on the normalized expression of six T cell marker genes: *CD3D, CD3E, CD3G, CD4, CD8A*, and *CD8B*. Next, we identified spots with significant local co-expression of two gene pairs: *IL1RL1*/*IL33* and *IL1RL1*/*FOXP3*. We used stlearn's within-spot analysis mode, which employs a permutation test to assess the statistical significance of co-expression signals. This process generated a co-expression score for each gene pair in every spot. Finally, to determine the spatial autocorrelation between T cell presence and gene co-expression, we performed a Bivariate Moran's I analysis. This test was performed separately to correlate the T cell module score with the *IL1RL1*/*IL33* co-expression score and the *IL1RL1*/*FOXP3* co-expression score. Using a significance cutoff of p < 0.05, spots were classified into one of four categories: 1) High co-expression/High T cell score; 2) High co-expression/Low T cell score; 3) Low co-expression/High T cell score; 4) Low co-expression/Low T cell score to represent the 4 types of T cell colocalization with co-expression of gene pairs *IL1RL1*/*IL33*and *IL1RL1*/*FOXP3*.

## CRediT authorship contribution statement

**Chenhao Zhou:** Writing – review & editing, Writing – original draft, Visualization, Methodology, Investigation, Formal analysis, Data curation, Conceptualization. **Jazmina Gonzalez Cruz:** Writing – review & editing, Supervision, Investigation, Data curation. **Siok Min Teoh:** Methodology, Investigation, Formal analysis, Data curation. **Minh Tran:** Methodology, Formal analysis. **Denise Goh:** Methodology, Formal analysis, Data curation. **Divyaa Narayanan:** Methodology, Formal analysis, Data curation. **Xiao Tan:** Writing – review & editing, Visualization, Formal analysis, Data curation. **Thi Viet Trinh Dang:** Methodology, Formal analysis, Data curation. **Zewen Kelvin Tuong:** Writing – review & editing, Supervision, Conceptualization. **Kevin Gillinder:** Methodology, Investigation, Formal analysis. **Abate Bashaw:** Methodology, Formal analysis, Data curation. **James W. Wells:** Writing – review & editing, Investigation, Conceptualization. **Ian H. Frazer:** Writing – review & editing, Supervision, Investigation, Funding acquisition, Conceptualization. **Quan Nguyen:** Supervision, Methodology, Data curation. **Yu Chen:** Writing – review & editing, Supervision, Investigation. **Meihua Yu:** Writing – review & editing, Writing – original draft, Supervision, Project administration, Methodology, Investigation, Formal analysis, Data curation, Conceptualization. **Janin Chandra:** Writing – review & editing, Writing – original draft, Visualization, Supervision, Project administration, Methodology, Investigation, Funding acquisition, Formal analysis, Data curation, Conceptualization.

## Funding statements

MY was supported by the 10.13039/501100000925National Health and Medical Research Council (NHMRC) Australia (APP1124265) and 10.13039/100014718National Natural Science Foundation of China grants (No. 32101146). YC is supported by International Collaboration Project of Chinese Academy of Sciences (GJHZ2072) and Shanghai Shuguang Program (21SG39). IHF is supported by the 10.13039/501100000925National Health and Medical Research Council (NHMRC) Australia (APP1173927), and the Merchant Foundation and the Metal Manufacturing Pty Ltd. JC is supported by the Garnette Passe Rodney Williams Memorial Foundation Mid-Career Fellowship and by the Zelman Cowan Academic Initiative.

## Declaration of competing interest

The authors declare that they have no conflict of interest.

## Data Availability

The accession number for the spatial transcriptomic dataset of K14E7 skin reported in this paper is GSE297947 (https://www.ncbi.nlm.nih.gov/geo/). The spatial transcriptomic dataset of human CIN3 samples was derived from UQ eSpace (https://espace.library.uq.edu.au/view/UQ:1bd2e07).

## Appendix A Supplementary data

Figure S1 ***Il33* and *Il1rl1* gene expression in K14E7 ear skin. (A)***Il33* and *Il1rl1* gene reads per million (RPM) of the whole ear pinnae skin of K14E7 and C57BL/6 mice, analysed by bulk RNA-seq. **(B, C)** Gene expression of *Il33* and *Il1rl1* from the epidermis and dermis of K14E7 and C57BL/6 mice using qPCR. Data are presented as means ± SD; ns p > 0.05, *p < 0.05, **p < 0.01, ***p < 0.001, analysed by unpaired two-tailed *t*-test.

Figure S2 **IL1RL1 protein expression is not detectable on myeloid cells in K14E7 skin.** Flow cytometry pilot experiment shows the plots of IL1RL1 protein expression on MHCII ^+^ CD103^+^ cDC1 and MHCII^+^CD301b^+^ DCs derived from K14E7 and *Il1rl1*^−/−^xK14E7 ear pinnae skin. Data are from one experiment (n = 1).
