# Supplementary material for: IL-33/IL1RL1 (ST2) signalling drives immune suppression in squamous intraepithelial hyperplasia
Source: Tumour Virus Res. 2026 Sep 1;22:200350. doi: 10.1016/j.tvr.2026.200350 (PMC13579448; doi:10.1016/j.tvr.2026.200350)
Supplement: Supplementary file 1 [file mmc1.pdf]

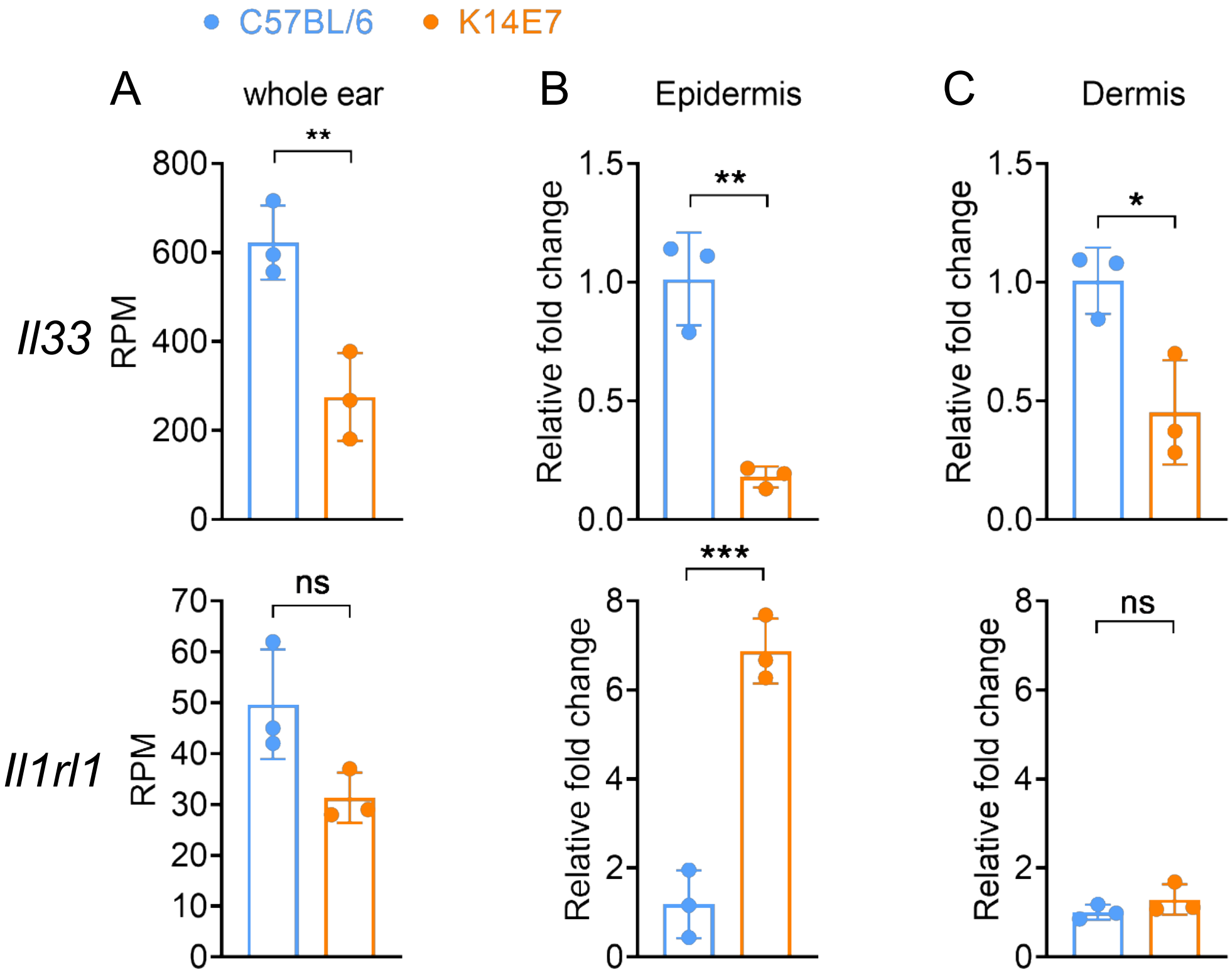

C57BL/6
K14E7
A
whole ear
B
Epidermis
C
Dermis
Il33
RPM
Relative fold change
**
**
*
Il1rl1
ns
***
ns
800
600
400
200
0
1.5
1.0
0.5
0.0
70
60
50
40
30
20
10
8
6
4
2
