# Supplementary material for: IL-33/IL1RL1 (ST2) signalling drives immune suppression in squamous intraepithelial hyperplasia
Source: Tumour Virus Res. 2026 Sep 1;22:200350. doi: 10.1016/j.tvr.2026.200350 (PMC13579448; doi:10.1016/j.tvr.2026.200350)
Supplement: Supplementary file 2 [file mmc2.pdf]

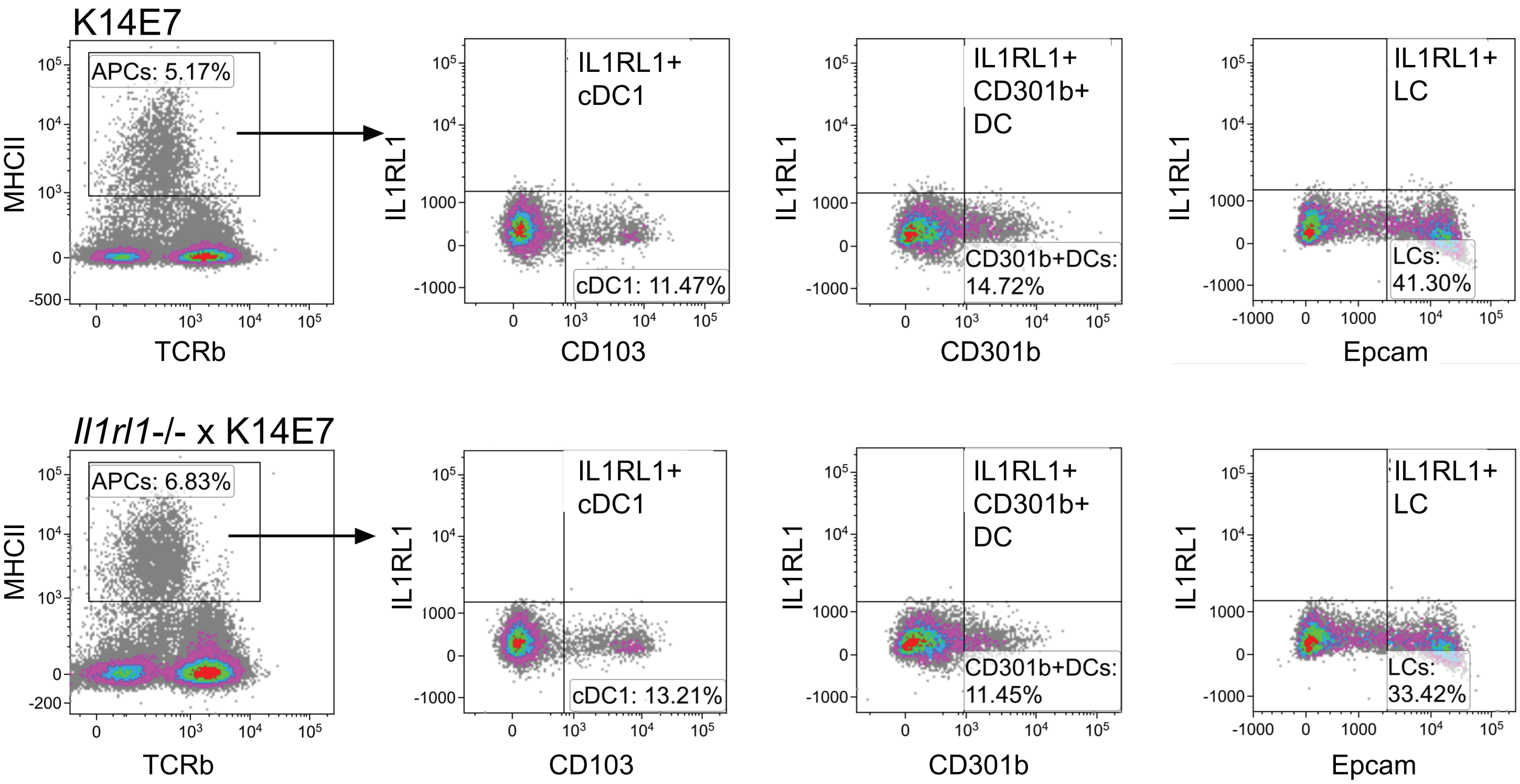

K14E7
APCs: 5.17%
MHCII
TCRb
IL1RL1+
cDC1
cDC1: 11.47%
IL1RL1
CD103
IL1RL1+
CD301b+
DC
CD301b+DCs:
14.72%
CD301b
IL1RL1+
LC
LCs:
41.30%
Epcam
Il1rl1-/- x K14E7
APCs: 6.83%
cDC1: 13.21%
CD301b+DCs:
11.45%
LCs:
33.42%
